# Advancing equity, diversity, and inclusion in the American College of Neuropsychopharmacology (ACNP): advances, challenges, and opportunities to accelerate progress

**DOI:** 10.1038/s41386-020-0784-y

**Published:** 2020-08-03

**Authors:** Jack E. Henningfield, Sherecce Fields, James C. Anthony, Lawrence S. Brown, Carlos A. Bolaños-Guzmán, Sandra D. Comer, Richard De La Garza, Debra Furr-Holden, Albert Garcia-Romeu, Dorothy K. Hatsukami, Armin Raznahan, Carlos A. Zarate

**Affiliations:** 1 PinneyAssociates and The Johns Hopkins University School of Medicine, 4800 Montgomery Lane, Suite 400, Bethesda, MD 20184 USA; 2 Department of Psychological & Brain Sciences, Texas A&M University, 4235 TAMU, College Station, TX 77843 USA; 3 Michigan State University, College of Human Medicine, 909 Wilson Road Room B601, East Lansing, MI 48824 USA; 4 START Treatment & Recovery Centers, 22 Chapel Street, Brooklyn, NY 11201 USA; 5 Columbia University Irving Medical Center and the New York State Psychiatric Institute, 1051 Riverside Drive #120, New York, NY 10032 USA; 6 University of New Mexico Health Sciences Center, College of Pharmacy, Albuquerque, NM 87131 USA; 7 Michigan State University, College of Human Medicine, 200 E First St., Flint, MI 48503 USA; 8 The Johns Hopkins University School of Medicine, 5510 Nathan Shock Drive, Baltimore, MD 21224 USA; 9 University of Minnesota Medical School and Masonic Cancer Center, 717 Delaware St SE, Minneapolis, MN 55414 USA; 10 National Institute of Mental Health, 6001 Executive Boulevard, Bethesda, MD 20892 USA

**Keywords:** Medical research, Neuroscience

## Abstract

It is increasingly accepted that higher levels of excellence and innovation in research can be achieved by organizations that promote equity, diversity, and inclusion across several domains including ethnicity and gender. The purpose of this commentary is to provide an overview of the methods used to increase diversity within ACNP, as well as recommendations for accelerating progress. Annual membership surveys confirm increases in female membership and leadership positions, slower but encouraging signals for “Asian” and “Hispanic” members, and less progress for African American and other ethnic populations. Meetings have become visibly more diverse, due in part to ethnic minority travel awards and apparently increasing diversity among guest attendees. Evidence of increasing inclusion includes well-attended networking events and minority-relevant programming, active communications about diversity-related events and resources, and strong statements by ACNP leadership that embrace diversity as a core value and support collaboration among key committees and task forces to identify and implement pro-inclusion and diversity-enhancing efforts. We believe ACNP can accelerate progress with more scientifically valid approaches to assessing diversity and inclusion. The current membership survey includes five outmoded ethnic options and postmeeting surveys that are not designed to assess inclusion efforts and consequences. Measures should be developed that better characterize diversity and assess efforts to reduce the barriers that exist for potential non-White populations (e.g., annual membership and meeting attendance costs). Increased collaboration with NIH and other organizations that are committed to these same goals may also contribute to acceleration of progress by ACNP and other scientific organizations.

## Introduction

In scientific organizations, the highest levels of excellence, innovation, and relevance are achieved when research teams incorporate deliberate policies of equity, inclusion, and diversity [1–6]. The National Institutes of Health (NIH) embraces such policies; its Office of Equity, Diversity, and Inclusion (OEDI), states “We cultivate a culture of inclusion where diverse talent is leveraged to advance health discovery” [7]. These principles have been employed by the American College of Neuropharmacology (ACNP) via membership outreach, annual ACNP meeting participation, and involvement in strategic planning for the future [8], as well as more recent efforts (Table 1). This commentary is based in part on poster presentations at the 2016 and 2018 annual meetings that addressed the trends in meeting participation, membership, and challenges to increasing inclusivity and diversity. The posters included informal polling of colleagues from ACNP and the College on Problems of Drug Dependence (CPDD) and formal survey data from ACNP and CPDD [9–11]. Presentation of the posters at ACNP and CPDD precipitated virtual focus groups at the meetings and postmeetings that were rich in thinking about the challenges to diversity and inclusivity and how to accelerate progress. Note that diversity enhancement efforts by ACNP have been focused on race and ethnicity and this is the main focus of this commentary, however, ACNP has been increasing its attention to gender identification and sexual orientation, as has NIH and so this is included. There are many other dimensions of diversity, including age and disabilities that are important to consider but beyond the scope of this commentary.

**Table 1:** Examples of diversity- and inclusivity-enhancing efforts by ACNP.

Inclusion- and diversity-enhancing efforts by ACNP in general include:
• Core value added to Website under “About Us”: “Commitment to Diversity: The College and its members actively promote diversity and inclusion within the College and within our field not limited to race, ethnicity, religion, disability, age, sex, gender identity, sexual orientation, intellectual perspectives and points of view.”
• Included objectives and goals related to inclusion and diversity in 2019 strategic plan.
• Collaborating task forces: diversity & inclusion (renamed from “Minority” in 2019), Latin American, Membership Advisory, and Women’s, which share ideas with various ACNP committees including Membership, Education and Training, the Strategic Planning Program, and the ACNP Council.
• Underrepresented minority (URM) resources on ACNP website @ https://acnp.org/education-opportunities/urm-resources/.
• Strong vocal support by ACNP presidents in communications to the membership and at meetings.
Efforts to foster inclusivity and diversity at the annual meeting include:
• Encouragement of submissions relevant to “improving diversity in the field of psychiatric neuroscience” in annual meeting program Call for Proposals.
• Explicit panel submission guideline statement: “Panel participants should include women, underrepresented minority (including LGBTQ+), and/or early career scientists/clinicians. A strong justification is required for proposals in which participant diversity is not included.”
• Networking events and luncheons.
• Diversity meeting events list emailed to members monthly premeeting and announced daily during meeting by email, Twitter, and Annual Meeting app.
• Travel awards for 2019 annual meeting: 62 total awards were equally divided by gender (male/female). Nineteen awardees (31%) self-identified as non-White (3 African American, 7 Asian, 9 Hispanic), and 43 as White.
• Mentoring program for all travel awardees.
• Encouragement of members to visit travel awardee posters.
• Mother’s room for nursing mothers and daily childcare are available for all meeting attendees, and provided without charge for current travel awardees, URM Past Travel Awardees, and Associate Members.
• Diversity pins including rainbow pins and “My preferred Pronouns are: “she-her,” “they-them,” “he-him,” “Pride,” and blank” for self-fill-in.
• All URM travel award applications are reviewed by chairs of the Diversity & Inclusion Task Force.
• Supplemental funding and meeting mentors provided to eligible URM Past Travel Awardees for 2 years post Award year. Also includes round-trip airfare, hotel accommodations, $50 per day meal stipend, waived registration fee, $80 per day childcare, and opportunity to present a poster.

This commentary provides a brief update on ACNP efforts to enhance equity, inclusion, and diversity, and ideas to accelerate progress. The authors believe that inclusion and diversity progress has been unacceptably slow but can be accelerated by building on ACNP’s recent efforts to better characterize diversity, understand barriers, and implement science guided approaches to addressing these systemic issues.

### The challenge of consensus and the value of differences and personal experiences

There are numerous challenges when scientific organizations try to promote equity, diversity, and inclusion. Members of ACNP and most scientific organizations vary widely in their self-identified ethnicity, race, ancestry, and life experiences, complicating efforts to reach consensus on approaches and priorities. Due to past and daily experiences, many scientists who do not identify as White question the genuineness of the commitment of predominantly White organizations which announce their intent to increase diversity. However, in the face of growing racial intolerance and violence in society, it is increasingly recognized that scientific organizations must do more to contribute to the promotion of inclusivity and diversity [12].

This commentary was developed during 2019 and early 2020 and submitted for publication on May 8, 2020. The authors feel that the national and global dialog on issues related to equity and inclusiveness in society precipitated by Mr. George Floyd’s death on May 25th increase the urgency of all individuals and organizations rethinking their approaches and their commitment to greater equity, inclusion, and diversity. ACNP responded to Mr. Floyd’s death on its website (www.acnp.org and at https://rdcu.be/b5EE3). We hope that this commentary will support ACNP’s efforts in past years including as discussed in its 2019 Strategic Plan.

Increasing equity and inclusion does not mean that organizations should be “color blind” and discount differences among populations. Color blindness is a sociological concept that embraces a society in which racial classification does not limit a person’s opportunities or result in different legal or societal treatment. Although a seemingly virtuous goal to some, it can also provide cover for discrimination and less overt, but no less destructive racial practices as discussed from a variety of perspectives by Crenshaw et al. [13]. Rather, we recommend recognizing and embracing the facts that ancestry, ethnicity, and life experiences are important to increasing the strength of scientific organizations [6, 9–11, 13].

### Why diversity and inclusion and what’s the difference?

Diversity and inclusivity are both mentioned in ACNP’s Core Value Commitment to Diversity (Table 1) and they are interrelated but not the same. In brief, diversity may be thought of as an objectively measurable goal or the “what,” whereas inclusivity is the “path” or the “why” and the “how” to achieve that goal. Inclusivity (aka “inclusion”) is related to the degree people feel welcomed and empowered, regardless of self-identified ethnicity, race, gender, sexual orientation, disabilities, and other characteristics [14, 15].

Organizations can set priorities and take action to enhance diversity (e.g., minority travel awards). Progress can be tracked by demographic surveys (e.g., self-reported gender, race, ethnicity, and ancestry). Inclusivity, on the other hand, is reflected by peoples’ perceptions, such as feeling “at home,” feeling free to openly express themselves, feeling that their ideas are valued, and that they can meaningfully contribute to the organization. These considerations are key for underrepresented people seeking to participate and sustain their participation in organizations, academic institutions, and businesses [7, 14–20].

Efforts to foster and assess diversity and inclusion should be closely coordinated lest efforts to advance one do not advance the other. For example, a five category “race” survey and binary gender survey approach may leave some people thinking that the organization does not understand or care about people who do not feel they fit into these categories. Such an approach may diminish the individual’s feelings that the organization is right for them if they feel that an option such as “other” or not answering the poll is most appropriate (see Table 2).

**Table 2:** Diversity of ACNP Members & Associate Members by ACNP survey categories from 2014 to 2019 based on membership renewal data showing the actual number and percentage of the membership for each year.

	2014		2015		2016		2017		2018		2019	
Ethnicity/race	No.	%	No.	%	No.	%	No.	%	No.	%	No.	%
White^a^	811	78.5	870	79.9	916	81.3	941	81.5	964	81.4	998	82.0
African American	7	0.7	7	0.6	9	0.8	11	1.0	13	1.1	16	1.3
Asian	77	7.5	87	8.0	92	8.2	103	8.9	111	9.4	113	9.3
US Pacific Islander	1	0.1	2	0.2	3	0.3	3	0.3	3	0.3	3	0.2
Hispanic	29	2.8	36	3.3	40	3.6	42	3.6	43	3.6	44	3.6
Native American	0	0	0	0	0	0	0	0	0	0.0	2	0.2
Other^b^	25	2.4	–	–	–	–	–	–	–	–	–	–
Blank	83	8.0	87	8.0	66	5.9	54	4.7	50	4.2	41	3.4
Gender												
Male	804	77.8	832	76.4	851	75.6	859	74.4	873	73.7	877	72.1
Female	229	22.2	257	23.6	275	24.4	295	25.6	311	26.3	340	27.9
Total membership	1033		1089		1126		1154		1184		1217	

^a^Whites calculated by subtracting reported non-Whites from total membership.

^b^The option to select “Other” was only provided in 2014.

### Beyond 5–6 category race metrics

Diversity assessment by outmoded racial categorizations, such as the 5–6 race categories used by most organizations in the USA, is problematic from many perspectives. Race has long been viewed by many as a social and political construct wrapped in a cloak of biology as discussed by DuBois in 1906 [21], and more recently NIH’s Human Genome Project which has made clear that racial categorizations are “also perpetuating misguided notions that discrete genetic groups exist” [22, 23]. This does not mean that “race-blind” approaches should ignore heritage and other variables potentially associated with differences in biology, disease risk, and risk of discrimination and hate crimes. Similarly, although “ethnicity” may be more informative regarding social, cultural, and regional heritage, it can be limited in biological research and medical practice [24].

NIH Director Francis Collins has concluded that “‘Race’ and ‘ethnicity’ are poorly defined terms that serve as flawed surrogates for multiple environmental and genetic factors in disease causation, including ancestral geographic origins, socioeconomic status, education and access to health care. Research must move beyond these weak and imperfect proxy relationships to define the more proximate factors that influence health” [25].

It is beyond the scope of the commentary to propose how ACNP should measure and characterize the diversity of its meeting participants and membership. There does, however, seem to be increasing consensus that a survey approach with binary assessment of gender and five race categories is (1) a flawed approach to assessing diversity, (2) out of step with the evolving science and not a good reflection on a leading neurobiological science organization, and (3) does not send a message of inclusion. Our general thinking aligns with the statement by Yudell et al. [24] related to race-based categorization: “It is time for biologists to find a better way.”

### Trends in ACNP diversification

The ACNP has evolved considerably since its earliest years in which, despite limitations of the data, women appeared to represent only a few percent of the membership and non-White members fewer still. Change and increased diversity have been enabled by changes in the organizational bylaws, membership procedures and extensive affirmative efforts to mentor, encourage, and include in meetings women and other URPop, and travel awards (Table 1).

Table 2 summarizes trends in diversity of ACNP members by sex and ethnicity from 2014 to 2019 as measured during online membership renewal. As shown in the table, 82% of the membership self-identifies as White. Diversity of annual meeting participants, which includes many nonmember guests, is not tracked. However, in our estimation the fraction of meeting attendees who might not identify as White has increased more rapidly than diversity in membership in recent years. Such a trend would not be surprising given the increasing numbers of URM travel awardees, minority focused symposia, and the noticeably increasing diverse pipeline of members’ trainees and nonmember scientists invited to meetings. These trends have the potential to contribute to ACNP inclusivity.

However, the numbers of respondents who did not respond or responded as “other” in Table 2 are troubling. These numbers may be influenced by some people who may check a category that they understand is supposed to represent them although they do not believe it really does. For example some people may select “Asian” despite misgivings about its application to them or they may select other or not respond because the operative US Federal government’s definition of Asian is very broad and lumps into the same “race” populations who do not feel they should be categorized as racially or ethnically the same [26]. Note that the census bureau definition that is the default definition is as follows: “A person having origins in any of the original peoples of the Far East, Southeast Asia, or the Indian subcontinent including, for example, Cambodia, China, India, Japan, Korea, Malaysia, Pakistan, the Philippine Islands, Thailand, and Vietnam” [27].

Increasing female meeting attendance, membership, and leadership has been ACNP’s greatest area of progress in diversification. The percentage of members who were women grew from about 7% in 1990 to about 28% (340) in 2019, with females representing 43% of 2019 meeting attendees. The ACNP Women’s Task Force report [28] provides more data and insights into factors contributing to this growth. One striking finding was the increasing overrepresentation of females serving on committees and in leadership positions over the past two decades as compared to their proportion as members. In fact, four of the ten presidents since 2010 (including 2020) are women; a striking increase given that the first female president (Dr. Eva Killam) served in 1988 with only three more elected prior to 2008. The 2020 president, Dr. Maria A. Oquendo, is ACNP’s first minority president and she will serve with a Council in which two of the six members are minority (Dr. Victoria Arango and Dr. Carlos Zarate). In 2020, Dr. Zarate was elected as president of ACNP. It also appears that new women members may account for a larger fraction of non-White members than new male members.

Table 2 shows that the percentages of the membership that identified as White, Asian, and Hispanic have steadily increased since 2014. Other non-White categories have shown little change.

In 2014, the only year that “Other” was an option, it constituted the third largest non-White category and no selection (“blank”) exceeded all non-White options. Interestingly, the absolute numbers and percentages of members who chose not to self-identify their ethnicity varied but overall declined over these 6 years to a level that was slightly lower than those who self-identified as Hispanic. The apparent fluidity in self-reporting suggests that the survey approach leaves some members without clearly appropriate options and that some survey respondents apparently change their response from year to year.

### Challenges and barriers

A 2016 informal survey of past and present members of ACNP’s Diversity & Inclusion Task Force, and members of the CPDD Underrepresented Populations (URPop) committee who attended ACNP meetings identified several impediments to efforts to increase interest and willingness to be nominated for membership in ACNP [9]. CPDD members were included because some were members of both organizations and all were frequent attendees of ACNP.

The financial cost with its enduring annual commitment received the highest score on the 0–9 rating scale—a finding that aligned with other analyses [29–31]. Becoming a member of ACNP and sustaining membership includes an annual financial commitment of at least $3000–$5000 given that membership renewal is $400, and annual meeting registration is $600 (2019 figures), in addition to per diem and travel costs that vary depending on where the meeting is held and where the member lives. According to the ACNP bylaws, members “who have three successive absences from the annual meetings will be referred by the Secretary to the Membership Committee… [which is] empowered to recommend to Council that nonparticipating members be terminated.” For members with consistent research support from NIH or employers, such expenses can be largely covered by grants. However, minority scientists are less likely to have consistent grant funding and salaries to cover such expenses during lapses in research funding because minorities receive less than 5% of NIH R01 awards [29–31] and tend to have lower incomes. Other factors seemed more related to inclusion, e.g., how welcoming the organization was perceived to be and a lower perceived importance of ACNP membership as compared to other organizations [9].

### There is a pipeline problem, but it need not prevent progress

Part of the challenge of increasing diversity in ACNP cuts across all areas of science, technology, engineering, and math in which it is believed that a broader pipeline of people interested in science will contribute to increased diversity across scientific disciplines [32, 33]. The pipeline metaphor has flaws and limitations, including the frequent assumption of a linear pathway from early education to scientific career, whereas it is frequently a nonlinear process with many potential pathways and points of entry to science careers [34–36]. On this premise, efforts to increase equity and inclusion that increase interest and opportunities for meeting attendance and membership would be expected to contribute to diversification. Furthermore, because the pipeline problem is a concern to many scientific organizations it would also seem mutually beneficial for ACNP and other scientific organizations to work together and learn together in efforts to increase interest and opportunities in science careers more generally. In particular, ACNP may seek increased opportunities to collaborate with the National Academies of Science, Medicine, and Engineering, NIH, and other scientific societies whose interests overlap with ACNP, including the CPDD, Society for Neuroscience, and the Society of Behavioral Psychiatry.

### Lessons learned and ideas for accelerating progress

Efforts to increase meeting inclusion and diversity need to be better coordinated with efforts to increase membership diversity. Efforts such as travel awards and sponsorship/support of minority persons by ACNP members summarized in Table 1 appear to increase meeting diversity with the caveat that meeting diversity has not been numerically tracked. Although this creates the opportunity for increased inclusion and ultimately membership diversity, it would be helpful to systematically study how the meeting experience actually affects attendees’ interest in becoming members.

Expert opinions from informal polls [e.g., 9, 10] and presented in task force meetings are useful, but we need scientifically valid survey instruments to track perceptions of inclusion and equity and the impact of efforts such as those summarized in Table 1. Informal discussions in the various networking events (opening reception, special luncheons and networking events, and poster sessions) by several of the co-authors of this commentary suggest that many of the visiting attendees feel positively about ACNP and may be interested in becoming members. Such anecdotal data suggest that ACNP would be well served by systematically collecting data to assess which of ACNP’s efforts appear to be inclusivity enhancing, what may be counterproductive, and what might be tried in the future. ACNP’s postmeeting surveys are helpful but not designed to achieve these ends.

The diversity of ACNP meeting attendance and membership are not captured by a binary male/female gender questionnaire and five category ethnicity/race questionnaire: a new survey approach is warranted. An expanded survey approach should include assessing the diversity of those attending annual meetings as well as membership to more realistically characterize ACNP’s diversity. The survey should leave no person feeling that they do not fit because none of the options apply to them.

Systematic assessment of inclusivity and diversity issues could likely benefit from consulting experts in this area, including NIH offices with relevant experience to ensure that it is socially sensitive and methodologically sound. For example, the NIH OEDI, the Sexual & Gender Minority Research Office, and the Office of Research on Women’s Health, and the National Institute on Drug Abuse (which presently contributes funding to ACNP’s inclusivity and diversity-enhancing efforts) have experts in diversity and inclusion.

An interim data-generating step that might be taken quickly would be to offer two additional options in the gender and “race” sections of the diversity questionnaire that is currently used by ACNP and many other scientific organizations: (1) “none of these describe me” along with an option to self-identify as the member believes is most appropriate, such as (2) “... better describes me.”

Scientifically sound data that are analyzed and presented to the standards expected for scientific publications are expected to be useful in supporting applications for funding for new inclusion and diversity initiatives from NIH and other organizations that may request evidence to evaluate progress. All of this is no small challenge, but considerable expertise is available within ACNP’s existing committees, the above-mentioned NIH offices, as well as from other experts and organizations [37–41].

Another proposal that seems likely to accelerate diversity in the near term would be to reduce the economic barriers to achieving and retaining membership. The informal data presented at ACNP [9, 10] suggests that many potential outstanding candidates for membership do not seek and reject offers of nominations because of the financial commitment of maintaining membership as discussed earlier. A proposal to reduce the financial barrier that was presented at the 2016 and 2019 ACNP meetings [9, 10] was to reduce the financial barrier as follows: qualified minority nominees for membership would be eligible for renewable 5-year awards for members, and 7 years for the term of associate members, to support membership dues, and meeting registration and travel expenses [9, 10]. Qualification criteria as well as the approach would need to be developed by appropriate ACNP committees with input from existing and potential minority members in the process of applying to NIH and/or private foundations for funding, and then would need to be evaluated to ensure that it can evolve over time as appropriate.

## Conclusion

The ACNP has made noteworthy progress in its efforts to become more inclusive and to accelerate diversification in membership and meeting attendance with support by public statements and actions of its leadership, which itself has shown promising signs of diversification. However, except for increasing female membership and small increases in Asian and Hispanic/Latino membership, diversity by other categories has shown little evidence of increase over the past decade. Moreover, although there have been efforts to increase inclusion and equity, there has been no systematic assessment of the impact of these efforts that might guide future efforts.

The 2019 Strategic Plan and earlier plans have set specific “Objectives & Strategies” related to inclusion and diversity, however, we believe that these should be revisited with consideration given to developing more specific diversity goals and timelines, along with a commitment to scientifically sound approaches to characterize diversity trends as well as perceptions of equity and inclusion. This should be done with application of the same scientific rigor and commitment that ACNP scientists bring to bear on other issues of import in their social and medical research. Collaborating with other scientific organizations with similar values and commitment may be mutually beneficial. Our experience indicates that the fundamental issues identified herein are not unique to ACNP but are shared by many scientific and medical organizations that may benefit from efforts underway at ACNP.

## Funding and disclosure

JEH: support for time and effort was provided by PinneyAssociates which provides consulting services on pharmaceutical and dietary product development and FDA regulated tobacco products; no funding or input was provided by commercial interests served by PinneyAssociates’ clients. JEH and all co-authors declared no conflicts related to the subject matter in this paper.
